# The Association of Health Literacy and Electronic Health Literacy With Self-Efficacy, Coping, and Caregiving Perceptions Among Carers of People With Dementia: Research Protocol for a Descriptive Correlational Study

**DOI:** 10.2196/resprot.8080

**Published:** 2017-11-13

**Authors:** Areti Efthymiou, Nicos Middleton, Andreas Charalambous, Evridiki Papastavrou

**Affiliations:** ^1^ Department of Nursing Faculty of Health Sciences Cyprus University of Technology Limassol Cyprus; ^2^ Department of Nursing Faculty of Health Sciences University of Turku Turku Finland

**Keywords:** health literacy, carers, dementia, ehealth

## Abstract

**Background:**

In the last decade, electronic health (eHealth) literacy has attracted the attention of the scientific community, as it is associated with the self-management of patients with chronic diseases and the quality and cost of care. It is estimated that 80% of people with chronic diseases are cared for at home by a family member, friend, or relative. Informal carers are susceptible to physical and mental health problems, as well as social and financial hardships. Nevertheless, there seems to be a research gap in terms of carers’ needs, skills, and available resources in the age of new technologies, with the vital role of eHealth literacy of the carers remaining unexplored.

**Objective:**

The aim of this study was to investigate the level of eHealth literacy and health literacy of primary and secondary carers of people with dementia, to explore the association between health and eHealth literacy, as well as their association with the caregiving variables: self-efficacy, coping, and caring perceptions.

**Methods:**

A sample of 200 primary carers (the carer who supports the people with dementia in everyday living) and 200 secondary carers (family member, friend, or other person in the social network assisting the primary carer in their role) will be recruited from dementia day care centers and Alzheimer’s associations in Greece and Cyprus. The study will be a cross-sectional correlational descriptive study. Tools to be used include the eHealth Literacy Scale adapted for carers to measure eHealth literacy, European Health Literacy Survey Questionnaire 16 (HLS-EU-Q16), Single Item Literacy Screener, Revised Scale for Caregiving Self-Efficacy, Carers of Older People in Europe (COPE) index for caregiving perceptions, and COPE brief to measure selected coping strategies. Descriptive statistics will be reported, and correlations between different variables will be explored with parametric and nonparametric measures.

**Results:**

As a preliminary study, the HLS-EU-Q16 has been validated in 107 older people. The internal consistency of the scale as estimated using Cronbach alpha coefficient was .77, somewhat lower than other validation studies. Recruitment of pilot study participants started in May 2017.

**Conclusions:**

Carers’ eHealth literacy is a new field. Whereas previous studies have focused on the role and impact of low eHealth literacy and health literacy among older adults, the eHealth literacy of carers, and in fact carers of people with dementia, has not been explored. We hypothesize an association between eHealth literacy and health literacy level with carers’ perceptions about caregiving role, self-efficacy, and coping strategies. A possible moderator in these associations is the secondary carers’ eHealth and health literacy level, which will also be explored. By confirming the above hypotheses, tailored eHealth literacy interventions for carers of people with dementia and their families will be developed as a direct outcome of this research.

## Introduction

### Carers and Internet Use

In the new digital era, new technologies are developed to support carers in their everyday role. However, most of the time, this is done without taking into consideration carers’ health literacy, digital skills, and electronic health (eHealth) literacy level. According to Eurocarers Association, “a carer is the person who provides unpaid care to someone with chronic illness, disability, or other long lasting health and care needs, outside a professional or formal framework.” People who provide care at least once or twice per week are those in the age range of 50 to 64 years, followed by the 35 to 49 age group according to the third European Quality of Life Survey [1]. In the case of carers of people with dementia, the age range is almost certainly older, as spouses and children older than 64 years are likely to become carers [2].

Older adults are considered to be the population group with the most difficulty in using new technology. In recent years, many studies have investigated the eHealth literacy of older adults, providing evidence that increased age and lower educational level are good predictors of lower eHealth literacy level and low Internet use [3-7]. Although there is vast literature about eHealth literacy in older adults **,** the level and the role of eHealth and health literacy among carers and, in particular, carers of people with dementia is very limited. There is, nevertheless, abundant information, mainly of descriptive nature, with regard to the type of Internet use among carers of people with different chronic diseases, without any further exploration or recommendation.

In a recent study in the United States, Kanthawala et al [8] investigated the type of questions and replies that people with diabetes and their carers post on the Web in the WebMD online diabetes website. People usually search information on their suggested treatment, questions that doctors have not replied to, and information on health habits. Most people consider the information on the Internet of good quality. Kanthawala et al classified questions searched into three categories: questions of fact, those related to policy or action, and those of value. Furthermore, they tried to address which type of resource is more adequate and clinically relevant for carers, concluding that community resources provided better quality results than search results of a common search engine such as Google.

In another study in the United Kingdom, Blackburn et al [9] explored the Internet use among 3014 carers. The study provided an overview of the digital gap among carers, which relates to both age as well as socioeconomic position. Half of the sample had never used the Internet. Of those using the Internet, 61% were frequent users (accessing the Internet once or more per week). Internet access by carers seems to be influenced by demographic and socioeconomic factors. Specifically, the age of the carer and the age of the patient, gender, employment status, living conditions, and hours of care are factors associated with Internet use. Similar findings have been reported by Kim [10] for a sample of carers of people with dementia. Specifically, younger carers (children and grandchildren), more educated, with a higher income, and fewer hours of caregiving are most likely to be health-related Internet users. Li [11] provided similar results for a sample of 812 carers of older adults.

According to Lam and Lam [12], the most common use of Internet among carers in Australia included chat sites and emails, indicating carers’ need to communicate. However, carers also used the Internet to retrieve information, as well as to access governmental services, for example, to pay bills. Interestingly, the study reported that carers who had been using the Internet 12 months before the study had better mental health in comparison with carers who had not used the Internet during that period. This is also supported by Kinnane and Milne [13] who have reviewed the literature for carers of cancer patients and have found that carers mostly use the Internet for information search for themselves or at a request by the cared for person for support group activity and email usage.

In a qualitative study, carers visiting a caregiving website mostly looked for health information and practical, legal, and financial issues. These preferences were directed by the type of caregiving. Kernisan et al [14] categorized replies in four categories: caring for parent, caring for self only, other caregiving situation, and unknown caregiving situation. In the case of carers of older people, practical issues were most frequently searched.

There is also a large number of studies looking at the effectiveness and the usability of Web-based support programs such as online communities, fora, and psychoeducational programs that aim to improve education and communication of carers [15]. A recent scoping review by Wasilewski et al [15] found that most studies mainly discuss carers’ experiences from participating in the programs or interventions, generally suggesting a positive attitude toward Web-based services. However, commonly no follow-up studies report either the usage and/or effectiveness of the specific interventions.

### Key Concepts and Their Associations Within the Proposed eHealth Literacy and Carers’ Research Framework

#### eHealth Literacy

As a term, eHealth literacy has gained considerable attention in recent years with the increased use of new technologies in health. Nevertheless, there is accumulating evidence that available technologies provided to people with chronic diseases or their carers are not properly used, or people are not using them because of lack of digital skills. In 2006, Norman and Skinner [16] presented the Lily model in an attempt to describe the different dimensions of eHealth literacy, defining the term as “the ability to seek, find, understand, and appraise health information from electronic sources and apply the knowledge gained to addressing or solving a health problem.” The Lily model refers to six basic types of eHealth literacy and categorizes them in two central types of skills: analytic- and context-specific skills. The analytic type includes:

1. Traditional literacy, which includes basic skills to read, understand, write, and speak language.
2. Information literacy, which describes the skills needed by a person to find, select, and use information available of any type.
3. Media literacy, which is defined as a process of metacognitive reflective strategies to place the information from several media sources in a social and political context.
4. Health literacy, for which several definitions have been used in the literature. One of the most frequently cited definitions is the one proposed by Ratzan and Parker [17], which refers to “The degree to which individuals have the capacity to obtain, process, and understand basic health information and services needed to make appropriate health decisions.” More recently, the construct of health literacy was explored in a cross-national European Health Literacy Survey among 8000 people from eight countries: Austria, Bulgaria, Germany, Greece, Ireland, the Netherlands, Poland and Spain. As a result, a new definition and conceptual framework was derived that incorporated elements from previous definitions, namely, “Health literacy is linked to literacy and entails people’s knowledge, motivation and competences to access, understand, appraise, and apply health information in order to make judgments and take decisions in everyday life concerning healthcare, disease prevention and health promotion to maintain or improve quality of life during the life course” [18].

In the context-specific type of skills, Norman and Skinner include (5) computer literacy, which is the ability to use computers, and (6) scientific literacy, which is the skill to understand the aims, methods, implementation, limitations, and politics of creating knowledge. As part of this theory, Norman and Skinner [19] developed the eHealth Literacy Scale (eHeals), one of the few and most frequently used tools to measure eHealth literacy.

Chan and Kaufman [20] have proposed a methodological and theoretical framework to analyze and measure eHealth literacy based on the Lily model and Bloom’s taxonomy. Bloom’s taxonomy describes the cognitive dimensions that are a prerequisite for any type of literacy and includes remembering, understanding, applying knowledge, analyzing, evaluating, and creating a coherent meaning. Furthermore, in their model, Chan and Kaufmann separated traditional literacy into three types: reading, writing, and numeracy.

Norman [21] discussed the need for eHealth literacy to be revised, taking into consideration the latest progress in Internet tools and environment with Web 2.0 and the use of social media and mobile Internet. Norman discusses the eHeals scale that had a good correlation with Web 1.0 and was tested with youth and youth workers, who were the frequent users during that period from 1990 to 2000. In 2011, the study of Van der Vaart et al [15] made the first critique to the model and the weak correlation between eHeals and Web 2.0, suggesting the revision of the tool.

After the revision of the Lily model, which actually included the cognitive factors of users, additional attempts to expand the model have taken place [22,23]. Gilstad [22] redefined eHealth literacy as “...the ability to identify and define a health problem, to communicate, seek, understand, appraise and apply eHealth information and welfare technologies in the cultural, social and situational frame and to use the knowledge critically in order to solve the health problem.” Four new dimensions were included to the Lily model: bodily experience (the ability to identify a health problem), procedural literacy (the “how” dimension of knowledge), contextual and cultural literacy (knowledge of a social situation: norms, values, rules, and regulations), and communicative expertise (the ability to convey personal health issues). Additionally, identifying the age bias toward young adults inherent to the Lily model and the eHeals questionnaire of Norman and Skinner, Koopman et al [23] considered dimensions that are relevant for older adults. The result was the Patient Readiness to Engage in Health Internet Technology instrument to measure the eHealth literacy of older adults.

More recent suggestions are the ones proposed by Norgaard et al [24] and Bautista [25]. Norgaard et al [24] have used concept mapping workshops with relevant stakeholders: information technology (IT) users, nonusers, patients, health care providers, and IT experts to update the dimensions contained in the eHealth literacy framework. Core dimensions that have been identified are the ability of info processing, a person’s motivation and interest in health and in using the digital services, feeling of safety and control, accessibility, sustainability, and appropriateness of Web-based services. Bautista [25] tried to redefine eHealth literacy as a term that “...involves the interplay of individual and social factors in the use of digital technologies to search, acquire, comprehend, appraise, communicate and apply health information in all contexts of healthcare with the goal of maintaining or improving the quality of life throughout the lifespan.”

#### Carers’ Self-Efficacy, Coping Strategies, and Social Support

Considering the important role that carers play for the national health systems, both the scientific community and policy makers alike have become more interested in maintaining carers’ health in recent years. Carers experience more stress than the general population, and they report higher use of antidepressants, are more susceptible to infections and cognitive decline, and have high mortality rates [26-28]. Furthermore, there are 3 close relatives for every person with Alzheimer disease [29]. For the purpose of this protocol, we will define the supporter relative or friend to the primary carers as the secondary carer. The term secondary carer is not a term regularly used; however, it has been previousy used in studies with carers of traumatic brain injury and cancer [30-32].

The stress process model [33] includes the core dimensions that influence carers’ well-being, mental and physical health, including concepts such as carers’ personality, primary stressors related to the severity of disease and perceived burden, secondary role strains, and secondary intrapsychic strains, including self-esteem, mastery, competence, and loss of self. According to Pearlin et al [34], self-esteem is influenced by four dimensions: role captivity, loss of self, competence, and gain. In caregiving, competence refers to the person’s ability to cope with the caregiving demands, and gain refers to the satisfaction that the carer might receive from caregiving tasks. Self efficacy and competence are often used interchangeably. Self-efficacy determines the various characteristics of a coping behavior; for example, when and if the coping strategy will be initiated, how long will it last, and the coping resources that will be used. Self-efficacy is influenced by “performance accomplishments, vicarious experiences, verbal persuasion, and psychological states” [35].

The coping strategies and the social support of the carer in combination with the different types of the stressors, according to Pearlin’s model, act as mediators of the mental and physical health of the carer [34].

Social support is included in Pearlin’s stress process model as part of the personal resources that are important to cope with life stressors [34]. The model has been subsequently adapted by Pearlin to conceptualize stress as a dynamic process with its origins in the social world. Economic and social position, as well as the neighborhood context plays a crucial role [36]. According to the convoy model of social relations, four types of networks are available: the diverse, family-focused, friend-focused, and restricted. The social convoy is actually the protective base of each person and is differentiated according to the specific structure (size, frequency, proximity of members, marital status, and participation in social organizations) and the quality of the relationships [37].

In the initial model by Pearlin [29], as part of the personal resources, aside from social support, there are also the coping strategies, including problem-focused, emotion-focused, and meaning-focused. According to Pearlin and Schooler [38], when a person has control over a role (ie, a family role), it is more effective to follow a problem-focused strategy. Where personal control over a role is lower (work and finances), the person may adopt emotion-focused or meaning-focused strategies when reappraising the situation. In some cases, there is the so-called compensatory coping, when after reappraisal, the person may proceed to a problem-focused strategy to reinvest [36].

Additionally, Lazarus and Folkman [39] distinguish within the transactional framework between coping processes and coping styles: the relationship between person and environment and the traits of the person, respectively. Part of the transactional framework is the appraisal theory **,** discussing the primary and secondary appraisal. In primary appraisal, the person focuses on the importance of the event, if it is irrelevant to their own well-being, benign, positive, or stressful. In the secondary appraisal, we encounter the contextual factor and the ability of the person to cope with the stressor.

**Figure 1:**
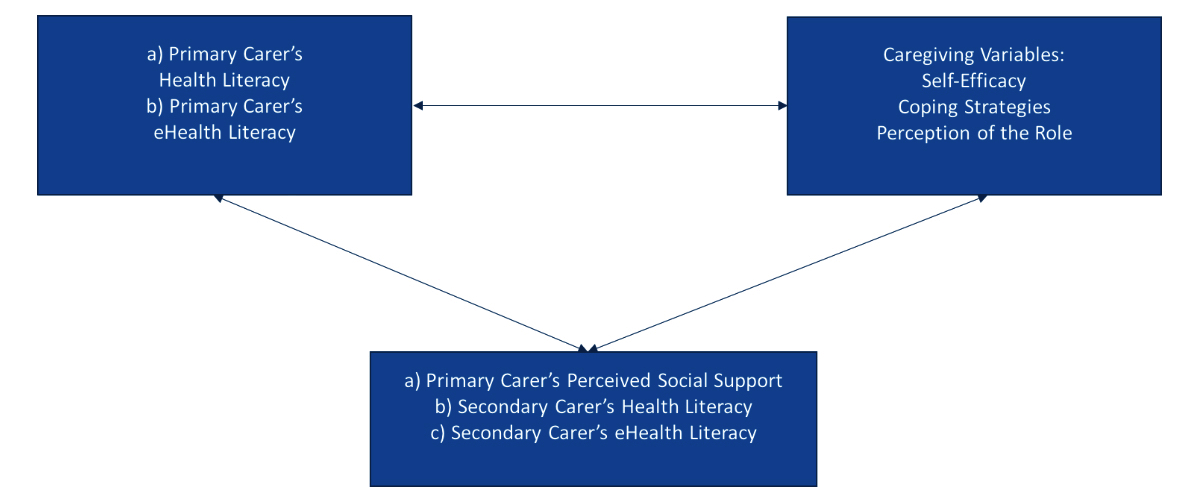
Health literacy and electronic health (ehealth) literacy of primary and secondary carer in association with primary carer’s perceived social support and the selected caregiving variables.jpg.

There is limited research on the associations between the abovementioned concepts, with research especially limited in terms of the role of eHealth literacy. Figure 1 connects the concepts in an effort to conceptualize the associations of health and eHealth literacy of primary and secondary carer and social support provided to the primary carer with self-efficacy, coping strategies, and perception of carer role.

In Figure 1, eHealth literacy is associated with health literacy, as described by Norman [16]. Taking into consideration the new definition provided by Soerensen et al [18], health literacy “entails people’s knowledge, motivation and competences to access, understand, appraise, and apply health information.”

Concerning the selected caregiving variables, self-efficacy is related to cognitive appraisal and acts as a motivator of action and selection of coping strategies [35]. Perceptions of carers’ role are related to coping strategies [40]. A person with enhanced self-efficacy is more likely to search for health awareness opportunities and feel empowered (being in control of one’s own health).

We presume the effect of health and eHealth literacy of the secondary carer and primary carer’s perceived social support on the health and eHealth literacy of the primary carer and the selected caregiving variables.

Social support is also a concept connected with health literacy, acting as a possible moderator in the relationship between low health literacy and poor health and is defined as “the degree to which individuals have access to social resources, in the form of relationships, on which they can rely” [41,42]. The support of social networks seems to play a role in the management of a person’s health problem and acts as a coping behavior. We can distinguish two types of social support: structural and functional. The structural support refers to the actual support network and as such the sources and extent of support as a result of the different roles that a person may have in the community (professional role, volunteering role, family role, and other roles). The social network a person belongs to may facilitate the communication of a health problem without directly improving health literacy but instead decrease the feeling of shame and possible stigma because of the inability to read and write about health information or seek medical advice for a health problem. Family and friends may also be facilitators in a decision about health or may take the decisions for the patient. This also may work in the opposite direction, where family and friends with low health literacy have a negative influence on the person’s health decisions [41].

The second dimension of social support, which may possibly interact with the level of health literacy, includes the emotional, informational, health reminder support, and tangible aspect of support and is referred to as functional support [41,43]. According to Lee [43], older adults with low health literacy had higher support concerning medical information and health reminder support. However, tangible support was rather low in this population with low health literacy, probably because of a lack of social networks [41,43].

### Aim of This Study

According to a recent review [15], we find a large number of Web-based support services for carers of people with dementia. Carers and new technologies is a topic of interest, so we consider it important to identify any issues related with carers’ health and eHealth literacy. Although there is some literature for older people or for carers of people with other chronic diseases, health and eHealth literacy have not been explored in carers of people with dementia. Furthermore, other than the primary carer, the role of health and eHealth literacy of the secondary carer will be assessed in this study. In addition, this study aims to explore the associations between health literacy, eHealth literacy and self-efficacy, coping strategies, social support, and caregiving perceptions of dementia carers, taking into consideration the role and support provided by the secondary carer.

As part of the study, health and eHealth literacy tools, as well as the Revised Scale for Caregiving Self-Efficacy will be validated in the Greek language for use among this population group.

The main research questions are

- RQ1a: What is the level of health literacy and eHealth literacy of dementia patients’ primary carers?
- RQ1b: What is the level of health literacy and eHealth literacy of dementia patients’ secondary carers?
- RQ2: Is there a difference between health literacy and eHealth literacy level of dementia patients’ primary and secondary carers, given the generation gap?
- RQ3: What is the association between health literacy and eHealth literacy of dementia patients’ primary and secondary carers?
- RQ4: What is the association (if any) between health literacy and eHealth literacy of dementia patients’ carers and caregiving self-efficacy?
- RQ5: What is the association (if any) between health literacy and eHealth literacy of dementia patients’ carers and their ability to cope with the stressors of caring?
- RQ6a: What is the association (if any) between health and eHealth literacy of dementia carers and their perceptions toward the caregiving role?
- RQ6b: What is the association (if any) of the health literacy and eHealth literacy of the dementia patients’ secondary carer and the primary carers’ self-efficacy, coping, and caregiving perceptions, and to what extent does the observed association between health literacy or eHealth literacy and caregiving variables in the primary carer differ according to the health and eHealth literacy of the secondary carer?
- RQ7: What is the association (if any) between social support and caregiving variables and to what extent the observed association between health literacy or eHealth literacy and caregiving variables in the primary carer differ according to the levels of social support?

## Methods

### Study Design

The study will be a cross-sectional correlational descriptive study design to explore the level of health literacy, eHealth literacy, and their association with caregiving self-efficacy, coping strategies, social support, quality of support, positive value, and negative impact of caregiving in Greece and Cyprus.

### Pilot Phase

Before the full scale research study, a pilot phase will be conducted to assess the appropriateness of selected questionnaires, the mode of data collection and length of interview, the acceptance of the research material by the primary and secondary carer, and expected challenges in sample recruitment. According to Connelly [44], the adequate number of people for a pilot study design is 10% of the total sample. Other researchers [45,46] suggest a number of 10 to 30. The minimum number of pilot participants in this case was set to a minimum of 17 to 30 primary carers.

### Sample

Carers of people with dementia will be recruited from dementia centers and Alzheimer’s associations in Greece, (Athens, Thessaloniki) and Cyprus. They will be invited to participate in the study following informed, signed consent. The sample will include primary carers (the carers who support the people with dementia in activities of daily living) and secondary carers (named family member, friend, or other person in the social network assisting the primary carer in their role). For each primary carer, a secondary carer who provides support to the primary carer will be identified. The secondary carer will be named by the primary carer as the closest person who supports the primary carer in his or her caring role. Selected questions will assist the primary carer to identify the supporter carers.

As there are many social cultural similarities related to caregiving between Greece and Cyprus, given the common language and historical and sociocultural background of both countries, it was decided to recruit one sample from both countries. Carers in both countries have the most important role in the care of people with dementia substituting for gaps in the national health care systems. The non-for-profit associations have undertaken the role of supporting and providing services to carers in Greece and Cyprus. In Greece, a number of services provided to carers by the not-for-profit associations are funded by the Ministry of Health through the mental health reform program [47].

Furthermore, the inclusion of two metropolitan cities from Greece, Athens and Thessaloniki, offers the opportunity to involve very active Alzheimer’s associations in Greece with both a high as well as more heterogeneous number of users, in an effort to achieve the inclusion of as wide as possible set of members from the target population in terms of their sociodemographic characteristics, as well as the variables of interest. As this is a correlational study, the multicenter convenience sampling aims to increase the observed variability in the variables of interest.

The sample size was calculated considering carers in Greece and Cyprus as one sample according to the above requirement The minimum required sample size with 95% power to detect a statistically significant correlation of the aforementioned variables of the magnitude of *r*=.25 (type I error 5%) is 168 primary carers and 168 secondary carers. To account for issues with possible inconsistencies in data, incomplete questionnaires, and missing values, it was decided to increase the recruitment to a sample of 200. Moreover, in this way, we ensure that the number of the secondary carers (and thus primary-secondary carers dyads) will not fall under the minimum required sample size, as it is likely that not all secondary carers may agree to participate. Estimated duration of the recruitment period will be 12 months.

### Recruitment Process

In Cyprus, prospective participants will be recruited from the Pancyprian Association of Alzheimer’s Disease and from the Alzheimer’s day centers, Ithaki, which are located in the city of Limassol and Pafos. We have selected these two day centers as they are currently the only services for carers. In Athens and Thessaloniki, recruitment will be done through the Alzheimer’s association. In Athens, there are currently six dementia day care centers: in the municipalities of Marousi (1), in Halandri (1), in the city of Athens (3), and in Ilioupoli (1). In Thessaloniki, there are two dementia centers. Furthermore, a sample will also be selected during the events on Carers’ day, which is usually organized by the associations annually.

Inclusion criteria for the primary carer include being a self-appointed carer of a person with dementia; supporting the person in activities of daily living, irrespective of the relationship with the person (spouse, children, sibling, friend, or neighbor); being over 18 years of age; and able to read and write in Greek.

The carers will be first approached by the manager of the centers and/or associations who will explain to them the aims of the study. If a carer fulfills the inclusion criteria and is willing to participate, she or he will be referred to the researcher for data collection.

Secondary carers will be nominated by the primary carer and will also be invited to participate in the study. The primary carer will initially contact the secondary carer asking if they are interested in participating, and the researchers will follow this communication to arrange the face-to-face or telephone survey interview.

The face-to-face surveys will be conducted at a place and time convenient for the primary carer. In the case of the secondary carer, an effort will be made to collect the data in face-to-face survey interviews, but the option for a telephone survey interview will be provided to reduce the likelihood of nonparticipation by the secondary carers. The primary carers will respond to the full questionnaire pack, whereas the secondary carers will be asked to respond to the health literacy and eHealth literacy scales (using the same tools as in the case of the primary carers), as well as providing information with regard to sociodemographic characteristics.

### Study Questionnaires

Information on sociodemographic characteristics will be collected from both the primary and secondary carers, as well as for the people with dementia they are caring for. Primary and secondary carers information will include age, gender, education, employment status, living situation, hours of care per week (primary carer), years of care (primary carer), number of care recipients (primary carer), relationship with the person with dementia (primary carer), care professional help (primary carer), relationship with primary carers (secondary carer), and type of support provided to primary carers (secondary carer). Information of the person with dementia will include age, gender, diagnosis, stage of the disease, and functional level.

### Health Literacy Measures

#### eHealth Literacy: eHeals Adapted for Dementia Carers in the Greek Language

eHeals, a self-report tool measuring eHealth literacy based on the Lily model, will be used [19]. The scale consists of 8 questions, and it assesses the users’ perceived skills at using health technology. In the original study, the scale showed good internal consistency with Cronbach alpha=.88. The eHealth scale taps into the usefulness, importance, perceived knowledge, and evaluation of Web-based health information, with a theoretical range for the overall score from 8 to 40. To date, the tool has been validated in Dutch [48], Italian [49], Chinese [50], and German [51] among varied population groups as school children, university students, and chronic disease patients. In the Dutch, Italian, and Chinese version, the questionnaire was treated as a unidimensional tool. In the German version, there were two dimensions (information-seeking and information appraisal). In all versions, the tools showed high internal consistency with Cronbach alpha ranging from .82 to .92 across the aforementioned studies. Only in the Dutch study were the participants people with rheumatic diseases, whereas the scale has not been previously used among carers of people with dementia.

For this study, the eHeals will be translated into the Greek language using backward-forward translation of the original English version. The questionnaire items will be adapted accordingly where necessary to address carers based on a review by an expert panel. The metric properties of the Greek version will be assessed using the Content Validity Index (CVI) based on the responses of an expert panel in the field of eHealth and health care to assess its content validity. Furthermore, the construct validity of the scale will be assessed in exploratory and confirmatory factor analyses as necessary. The internal consistency of the scale will be assessed using Cronbach alpha coefficient. The validation will be part of the analysis of data derived from the final sample.

#### The Internet Use Carers Profile

The Internet use carers profile will be measured using a series of 10 questions that assess the frequency and type of use, for example, use of websites, emails, e-learning, social media, interactive services, forums, blogs, mobile, and the Internet. It was deemed important to supplement the eHeals scale with these profile questions, as there has been much criticism with regard to the lack of relevant questions in the eHeals scale, given the Web evolution during the last decade [21,48].

#### European Health Literacy Survey Questionnaire 16 (HLS-EU-Q16) Short Form

In addition to eHealth literacy, the health literacy of the primary and secondary carers will be assessed using the European Health Literacy Survey Questionnaire 16 **(**HLS-EU-Q16) [52,53]. The long form of the questionnaire consists of 47 questions, whereas there are also two shorter forms, one with 16 and one with 6 questions. Due to the large number of questionnaires included in this study, it was decided to use the 16-item short form of the scale. The short form was developed based on Rasch modeling and is considered one-dimensional and discriminates three levels of literacy: sufficient health literacy, problematic health literacy, and inadequate health literacy. The tool has been validated in German [54,55], Bulgarian [53], Dutch [53], Israeli [56], and Swedish [57]. As far as we are aware, there is no published validation in Greek, even though Greece participated in the original cross-national survey.

#### Single Item Literacy Screener (SILS)

Single Item Literacy Screener (SILS) assesses inadequate health literacy and together with the HLS-EU-Q16 provides the information on the health literacy level of the study participants. SILS has been part of 16 questions developed by Chew et al [58]. Initially, 3 questions were identified as better predictors of low health literacy and difficulty in reading printed material. Chew et al [59] proceeded in selecting the single item (SILS) that had better sensitivity (ie, 39% at a score <2) and specificity (93%) than the other 2 questions in predicting inadequate health literacy. The question “How often do you need to have someone help you when you read instructions, pamphlets, or other written material from your doctor or pharmacy?” is replied with a 5-point Likert scale from 1=never to 5=always. A score of 2 and above is considered adequate health literacy level. SILS according to Brice et al [60] does not assess marginal literacy accurately, as it is defined based on the Short Test of Functional Health Literacy in Adults (S-TOFHLA): the person “has difficulty in reading and interpreting health texts.” SILS is easy to use in a clinical setting for a quick screening of health literacy, can discriminate between inadequate and adequate reading ability, and predicts well S-TOFHLA scores of low health literacy. For this specific study, SILS will be validated in Greek to assess the sensitivity and the specificity of the question and adjust the selected cut-off score for this specific population.

### Other Constructs (Dependent Variables)

#### Revised Scale for Caregiving Self-Efficacy

The scale assesses the self-efficacy of carers [61]. It consists of 15 items organized in three subscales, namely, (1) self-efficacy for obtaining respite, (2) self-efficacy for responding to disruptive patient behaviors, and (3) self-efficacy for controlling upsetting thoughts about caregiving. Internal consistency of the three scales was high with Cronbach alpha over .80. The Revised Scale for Caregiving Self-Efficacy has high correlation with depression, anxiety, anger, and social support scales [57]. This scale will be validated in Greek.

#### Perceptions Toward Caring: COPE Index

COPE index measures carers’ perceptions toward positive and negative values of caring [62]. It consists of 15 items and is part of a study protocol realized in five countries: Italy, Greece, Poland, Sweden, and the United Kingdom. Positive value of caring includes five items, and negative values includes six items. Furthermore, three additional items measure the quality of support, and one item taps into the financial hardships. Negative values items had high internal consistency (Cronbach alpha=.88) in comparison with positive values items with a more modest internal consistency (Cronbach alpha=.67). The criterion validity of the scale was assessed with the use of General Health Questionnaire, Hospital and Depression Scale, and World Health Organization Quality of Life-BREF [62]. Negative values items had significant association with all measures in all countries. Positive values of caring items demonstrated significant association with all measures but was restricted to certain countries (Sweden and Greece).

#### Brief COPE

Brief COPE assesses the coping strategies adopted by carers [63]. It consists of 28 items organized in pairs in 14 groups of strategies, namely, acceptance, active coping, positive reframing, planning, use of instrumental support, use of emotional support, behavioral disengagement, self-distraction, self-blame, humor, denial, religion, venting, and substance use.

#### Multidimensional Scale of Perceived Social Support—MSPSS

Multidimensional scale of perceived social support consists of 12 items measuring social network support, including three factors: significant other, family, and friends. The items are scored on a Likert scale from 1 (very strongly disagree) to 6 (very strongly agree) [64,65]. The higher score is 84 and, commonly, a cut-off score of 65 is used. The scale has been tested among different population groups from students to older adults, including patients with chronic diseases. High internal consistency was reported for overall scale (Cronbach alpha=.88), as well as for the subscales (significant other Cronbach alpha=.72, family Cronbach alpha=.85, and friends Cronbach alpha =.75) [63].

### Statistical Analysis

Descriptive statistics will be reported, and bivariate correlations between all variables of interest will be explored with parametric and nonparametric measures. Sociodemographic correlates associated with health literacy will also be assessed. Additional data analysis (eg, *t* test, analysis of variance) will be used as needed, for example, to investigate differences in eHealth and health literacy according to sociodemographic characteristics of the participants. The association between dependent variables (coping, self-efficacy, and caregiving perceptions) and independent variables (health literacy, eHealth literacy of primary and secondary carers) will be assessed in multiple regression models before and after adjusting for sociodemographic variables. The extent to which the observed association between health literacy and coping and caregiving perceptions among primary carers differs according to self-efficacy, social support, and the secondary carer’s eHealth and health literacy (moderators) will also be explored.

Concerning the adaptation and validation of the health literacy questionnaire, SILS, and Revised Scale of Caregiving Self Efficacy, face and content validity will be assessed by an expert panel. The metric properties (construct validity and internal consistency) will be assessed using exploratory and confirmatory factor analyses and internal consistency reliability analysis. Analysis will be performed by Statistical package for the Social Sciences (SPSS) version 22 (IBM Corp) and exploratory factor analyses with SPSS AMOS.

### Ethics Approval

Permission to conduct the study was granted by the National Committee of Bioethics in Cyprus on January 10, 2017, according to the National Law (EEBK ΕΠ 2016.01.151). The commissioner of personal data protection in Cyprus has been notified accordingly and confirmed notification on December 19, 2016 (study number 3.28.460). In Greece, the scientific committee of the Athens Association of Alzheimer’s Disease and Related Disorders have also been notified and approved the study on March 17, 2017, with a decision by the Executive Board. This process will be repeated for the Alzheimer’s association in Thessaloniki.

All participants will be fully informed about the purpose and the requirements of participation in the study. Consent forms will be signed, and participants will have the right to withdraw at any time. Confidentiality of the participants will be respected. Researchers will safeguard the well-being of the participants during the data collection.

Participants who are interested in receiving feedback will be contacted by email or telephone as soon as the results are analyzed and drafted. Researchers will try to make the participants feel comfortable and resolve any kind of conflict concerning the time, the place of the meetings, and the way that the secondary carers will be contacted.

To safeguard personal sensitive data, a database protected by a password will be developed and will be stored by the research team university computers. Only members of the research team will have access to the database. Hard copies of all measurements will be stored and locked in the Office of the Scientific Supervisor.

## Results

The pilot phase of the study is in progress. In the following section, we report some preliminary results of the validation of HLS-EU-Q16 in Greek for the purposes of this protocol.

A convenience sample of 107 older people from an outpatients’ eye clinic in Cyprus and open clubs for leisure activities for older people in Athens, Greece, participated in the validation of the scale (Table 1).

The internal consistency of the scale as estimated using Cronbach alpha coefficient was .77 and was adequate, even though it was somewhat lower that the respective figure observed in validation studies elsewhere. CVI for each item, as well as the overall scale was also calculated with a panel of experts (N=6) and a panel of health professionals (N=20), providing high scores for item-level CVI and scale-level CVI/average (S-CVI/Ave) in both groups. S-CVI/universal agreement (S-CVI/UA) was lower among health professionals compared with the group of experts (Table 2).

In-depth analysis of the results derived by the validation of HLS-EU-Q16 will be presented in a subsequent paper. The data collection of the pilot study started in May 2017, and the data collection for the main study is projected to start in October or November 2017.

**Table 1:** Sociodemographic characteristics of the participants to Health Literacy Scale-Europe-Questionnaire 16 (HLS-EU-Q16) validation.

Characteristics		n (%)
**Gender**		
	Women	62 (57.9)
	Men	45 (42.1)
	Total	107 (100)
**Age in years**		
	<60	9 (8.4)
	61-80	80 (74.8)
	>81	18 (16.8)
**Education**		
	No primary education	9 (8.4)
	Primary education	47 (43.9)
	Secondary education	40 (37.4)
	Tertiary education	11 (10.3)
**Profession**		
	Pensioner	84 (78.5)
	Employed	12 (11.2)
	Unemployed	2 (1.9)
	Other (eg, housekeeping)	9 (8.4)
**Family status**		
	Married	82 (76.6)
	Single	3 (2.8)
	Divorced	2 (1.9)
	Widowed	18 (16.8)
	Other	1 (0.9)
**Comprehensive health literacy level**		
	Sufficient	49 (45.8)
	Problematic	49 (45.8)
	Inadequate	9 (8.4)
**Health perception**		
	Good	77 (72)
	Neither good or bad	26 (24.3)
	Bad	4 (3.7)
**Quality of life perception**		
	Good	84 (78.5)
	Neither good or bad	20 (18.7)
	Bad	3 (2.8)
**Chronic illness**		
	Yes	58 (54.2)
	No	49 (45.8)
**Country**		
	Cyprus	69 (64.5)
	Greece	38 (35.5)

**Table 2:** Content validity index analysis of the European Health Literacy Survey Questionnaire 16 (HLS-EU-Q16).

Panel	Mean I-CVI^a^	S-CVI/Ave^b^	S-CVI/UA^c^
Group of experts (N=6)	.96	.96	.81
Group of health professionals (N=20)	.97	.97	.69
Total	.93	.97	.63

^a^I-CVI: item-level content validity index.

^b^S-CVI/Ave: single-level content validity index/average.

^c^S-CVI/UA: single-level content validity index/universal agreement.

## Discussion

### Principal Findings

In this study protocol, we have presented the preliminary results of the HLS-EU-Q16 validation. The validation was carried out among 107 older people in Greece and Cyprus, providing information for the comprehensive health literacy level of older people in these two countries. The main study will investigate the relationship of eHealth literacy and health literacy with caregiving self efficacy, coping strategies, and care management perceptions of carers of people with dementia. Previous studies have explored the associations between health literacy and coping strategies, health literacy and self-efficacy, coping strategies and care management, caregiving and self-efficacy, social support and self-efficacy, and social support and health literacy in different target groups. However, no previous study has adopted a unified approach or explored these issues in carers of people with dementia [36,66-69]. Furthermore, studies commonly focus on the primary carer. In this study, information will be also collected from the supporter carer (or secondary carer). The support provided by the secondary carer to the primary carer may influence the primary carer’s self efficacy, coping strategies, and/or caregiving perception. Furthermore, the health and eHealth literacy of the secondary carer may influence both the health and eHealth literacy of the primary carer, as well as acting as a moderator in the association between health literacy and caregiving variables in the primary carer.

eHealth literacy is a rather underresearched concept among this population, taking into consideration the age of the majority of carers (above 50 years). The idea of connecting eHealth literacy with caregiving becomes more challenging. New technologies are a core part of everyday life for a large percentage of the population worldwide, but still there are specific groups with low access to technological advances. Low income, low socioeconomic status, and racial or ethnic minorities are considered a predictor of Internet nonuse [5].

Carers and especially spouses could be considered to be a minority in the use of technology. On the other hand, several projects are funded to develop technological innovations to support carers in their role, including Web-based psychoeducational programs and support groups [70-73], interactive services (forums, online communities) [74-76], interventions for depression and stress management [77], e-learning courses and carer platforms or websites [76,78-81], telemedicine, and telehealth (global positioning system, sensor technologies) [82,83]. The need to investigate the level of eHealth literacy and related skills and resources in this population becomes more important considering the possible discrepancy between the development of new technologies for carers on the one hand and the actual frequency of use, and thus benefit, of such technology.

This is also confirmed by the systematic review by Chi et al [84]. Six types of technology-based interventions for carers were identified:

1. Education using mainly telephone-based, Web-based, and video interventions
2. Consultation using videoconferencing
3. Psychosocial or cognitive behavioral therapy intervention using telephone and videoconferencing tools
4. Social support using videoconferencing tools
5. Data collection or monitoring, including response center, sensors, and fall detectors
6. Clinical care delivery using videoconferences

Taking into consideration the large amount of research available on the usability and feasibility of this type of research, it is interesting that there is little focus on the skills required by this target population to use the aforementioned services.

### Limitations and Strengths

The challenges of this study concern the recruitment of carers, both in terms of access (hence a convenient sample of people in contact with services), as well as the time requirements and other elements of the recruitment procedure, mainly the survey completion time (estimated at 60 min) and potential difficulties in contacting and recruiting secondary carers. We expect that the majority of secondary carers will be the children or friends of the primary carer, making the arrangement of the survey interview challenging both in terms of time and location but also in terms of motivation to participate.

This study presents numerous strengths. Even though a convenience sample will be recruited, the recruitment will be from a variety of settings to increase the heterogeneity of the sample in terms of their sociodemographic characteristics, as well as the variables of interest. Furthermore, the eHeals questionnaire will be adapted to the needs of carers, and the HLS-EU-Q16 will be used and validated for the first time in this specific population. More importantly, the study will assess the level of health and eHealth literacy of Greek and Cypriot carers of people with dementia for the first time, as well as explore the role of these constructs in the caregiving process. This has important implications about the services provided.

Moreover, screening tools will be available to measure health and eHealth literacy levels for this specific population, and future research on eHealth literacy training of carers in Greece and Cyprus will follow.

**Conclusions**

Taking into consideration the fast technological progress, the demand for Web-based training and eHealth literacy training is only a matter of time. More and more resources are being developed to support carers on the Web, and the use and assessment of this type of technologies by carers are becoming essential skills that in future years will become obligatory. Focusing on training and developing, training classes and e-learning courses could facilitate the development of these specific skills among this population. Furthermore, the usage of new technologies and the Internet could act as a facilitator in the caregiving demands of carers.

## Abbreviations

COPE: Carers of Older People in Europe
CVI: content validity index
eHeals: eHealth Literacy Scale
eHealth: electronic health
HLS-EU-Q16: European Health Literacy Survey Questionnaire 16
I-CVI: item level content validity index
IT: information technology
S-CVI/Ave: scale level content validity index/average
S-CVI/UA: scale level content validity index/ universal agreement
SILS: Single Item Literacy Screener
S-TOFHLA: Short Test of Functional Health Literacy in Adults
